# Addiction specialists' perspectives on digital contingency management and its role within UK drug and alcohol services: A qualitative exploration

**DOI:** 10.1111/dar.14046

**Published:** 2025-03-18

**Authors:** Carol‐Ann Getty, Nicola Metrebian, Joanne Neale, Tim Weaver, John Strang

**Affiliations:** ^1^ National Addiction Centre, Institute of Psychiatry, Psychology and Neuroscience King's College London London UK; ^2^ Department of Mental Health & Social Work, Faculty of Health, Social Care and Education Middlesex University London UK

**Keywords:** addiction specialists, contingency management, qualitative, technology

## Abstract

**Introduction:**

Contingency management (CM), based on the principles of operant conditioning, uses positive reinforcement to promote behaviour change in individuals with substance use disorder. Research on CM has grown exponentially, with technology being used to expand the reach and scope of these interventions. The views of policy professionals and treatment providers on the remote delivery of CM are likely to play an important role in the development and application of these interventions.

**Methods:**

Semi‐structured qualitative interviews, analysed using framework analysis, were conducted with 22 UK‐based addiction specialists to explore their views on digital CM, including its place within UK drug and alcohol services and future developments. Participants included commissioners, policy professionals and clinicians.

**Results:**

CM was widely acknowledged as an effective, scientifically grounded and appropriate treatment approach for drug treatment and recovery. While addiction specialists see CM as a powerful tool in a comprehensive addiction treatment toolkit, they identify the barriers impeding its implementation, including a lack of awareness among treatment providers, commissioning challenges, resource constraints and ethical concerns. Remote delivery of CM was considered a promising approach for overcoming some of these barriers and enhancing CM delivery and engagement.

**Discussion and Conclusions:**

Technology needs to be integrated into clinical practice to expand the reach of treatment. While current evidence supports digital CM, concerns about digital literacy, technological barriers, resource constraints, public acceptability and political hesitancy highlight the need for further research to validate its feasibility and to explore the extent to which it should complement, rather than replace, in‐person treatment options.

## 1 INTRODUCTION

Contingency management (CM) is a behavioural intervention involving the adjunctive application of positive reinforcement (e.g. monetary incentives) contingent upon evidence of positive behaviour change. CM is based on the theoretical principles of operant conditioning and is among the most efficacious psychosocial interventions in promoting substance use‐related behaviours, including abstinence from smoking, alcohol and illicit drugs [1, 2, 3, 4, 5, 6, 7], medication adherence [8, 9], vaccination uptake [10] and attendance [11]. Despite CM's well‐established research evidence base and the UK's National Institute for Health and Care Excellence's recommendation to evaluate and integrate it into addiction services, few UK addiction specialists have experience with CM or implement it within their practice. Implementation has been hindered in part due to barriers, such as a lack of resources and concerns among treatment providers. The key challenge is in adapting CM interventions to overcome the known barriers to implementation while maintaining fidelity to CM's core principles.

One possible way to enhance access to CM while minimising the burden on resources and staff is to deliver it remotely using technology (e.g. mobile telephone text messaging services and apps, tablets and computers, pill dispensers, breath alcohol and carbon monoxide monitoring devices) [12, 13]. Theoretically, innovations in technology might allow CM to be implemented with enhanced fidelity and at a lower cost without compromising effectiveness while overcoming the need for frequent attendance at drug and alcohol services for in‐person monitoring, which is difficult to incorporate into routine treatment practice in the UK. Remote technologies have been integrated into CM interventions to objectively monitor the target behaviour of the intervention and act as a system enabling the delivery of the reinforcement when the target behaviour is achieved. A meta‐analysis of mobile telephone‐delivered CM (mCM) [14] and more recent evaluation studies [15, 16, 17, 18, 19, 20] suggest that these interventions are effective in generating positive behaviour change.

Previous studies of treatment providers have highlighted potential barriers to the adoption of CM, including a lack of awareness of CM and its effectiveness; concerns regarding the longevity of change; philosophical and ethical objections; public and political sensitivities and concerns that CM targeting substance use tends to re‐enforce abstinence as the goal of treatment as opposed to harm reduction [21, 22, 23, 24]. As CM typically requires frequent and close monitoring of the target behaviour, these interventions are sometimes considered too resource‐intensive to be practical and incorporated into routine clinical practice. Despite these concerns, most treatment providers supported the potential of implementing CM as part of a toolkit in adjunct to existing treatment and argued for a pragmatic approach to be taken: ‘if it works, use it’ [22].

Research on CM has grown exponentially over the last decade, with remote technologies being leveraged more than ever to expand the reach and scope of these interventions [25]. This coincides with technological innovations in the delivery of healthcare interventions in general but has intensified due to the COVID‐19 pandemic and public health control measures [26, 27]. It is therefore timely and essential for researchers to consider not only current perspectives on CM as a treatment approach more generally but also views on the use of technology to optimise and facilitate its delivery. No existing research has explored the views of policymakers and treatment providers on digital CM, although these would likely play an important role in the development and application of these interventions.

This qualitative study aims to ascertain addiction specialists' views on mCM, including its place within UK drug and alcohol services. Stakeholders play a significant role in the coordination, resourcing and delivery of services and therefore their views are imperative in ensuring that treatment approaches are responsive to the needs of those directly affected while promoting better resource allocation, public support and a comprehensive approach to treatment. Specific objectives were to explore participant's views on:

- The needs and expectations of CM as an approach to modify treatment‐related behaviours, including cessation or reduction, vaccination uptake, attendance and medication adherence.
- The use of technology devices to expand the reach of CM and the appropriateness of monitoring behaviour and delivering reinforcement remotely.
- Barriers to delivering mCM and strategies to overcome these.
- The client group(s) that might benefit the most from mCM.
- Future directions for mCM interventions.

## 2 METHODS

### 2.1 Participants

Participants were 22 key informants,^1^ each of whom was a specialist in the addiction field. They were all in senior positions in the UK and, between them, covered a range of professional disciplines and a range of agencies or organisations. Across the 22 participants, they included: (i) commissioners responsible for funding allocations and commissioning of drug and alcohol treatment (*n* = 5); (ii) policy professionals involved in making decisions about the implementation and delivery of addiction treatment at local or national levels (*n* = 2); and (iii) clinicians involved in the management or delivery of addiction treatment (e.g. senior university addictions academics (*n* = 5), addictions psychiatrists (*n* = 9), addiction‐specialist clinical psychologists (*n* = 2), addiction‐specialist GPs (*n* = 1), addiction‐specialist public health (*n* = 1), addiction‐specialist senior pharmacists (*n* = 2) and addiction‐specialist senior nurses or nurse‐prescribers (*n* = 2)), (N.B. totals exceed the number of participants as several participants met more than one criterion).

### 2.2 Sampling strategy

Expert sampling was used initially to recruit participants from an existing informal ‘Expert’ group established by JS during the COVID lockdown to provide senior peer support and enable consideration of COVID‐triggered operational adaptations. The ‘Expert’ group consisted of 19 members holding clinical and policy informant positions. The group founder disseminated the participant information sheet among group members and sought consent to contact them via email. The researcher (CAG) contacted willing participants to arrange the interview and obtain written consent. Using the snowball sampling technique, after each interview, participants nominated potentially eligible candidates to take part. For this sample, 14 were recruited from the ‘Expert’ group and 8 were recruited by snowballing.

### 2.3 Data collection

Semi‐structured interviews were undertaken by the post‐doctoral researcher (CAG) between May and August 2021. Although participants were likely to know about CM, awareness of how technologies could be leveraged to deliver CM interventions was likely to be uneven. To ensure in‐depth discussion around the novel advances in CM delivery could be generated, participants were provided with a summary sheet in advance. This provided information on the theoretical underpinnings of CM, how CM has been used to promote treatment‐related behaviours, how mobile technologies can be integrated into CM interventions and the state of the evidence base. Due to COVID‐19 restrictions at the time, interviews were conducted remotely using Microsoft Teams. Interviews lasted for 47 min on average (ranging from 26 to 62 min). Interviews were guided by a semi‐structured topic guide (Appendix A) and were recorded and transcribed automatically by Microsoft Teams. Transcripts were downloaded at the end of each interview, checked and edited for accuracy.

### 2.4 Data coding and analysis

The analytical method to analyse the interview data was Framework analysis [28], commonly used in applied research. Data coding and analyses were conducted in stages by CAG. A coding frame was developed, comprising a‐priori conceptual codes supplemented by inductive codes emerging from the data. A‐priori conceptual codes, based on previous literature and the CM theoretical framework, facilitated a clear progression from research aims to conclusions. Inductive codes were added as new themes emerged. NVivo was used for line‐by‐line transcript review, identifying and grouping key issues or concepts into coding folders. The coding scheme contained a total of 57 codes (Appendix B) that were applied consistently across all the transcripts. On review of the content of these codes, we consolidated them into main themes presented here under broad categories. Using framework analysis, a matrix was created, with participants as rows and themes as columns. Data were summarised and presented under each theme. This structured approach facilitated comparison across cases and allowed for nuanced insights and interpretations. The main themes and findings were discussed with the co‐authors who have expertise in substance use research and policy.

### 2.5 Ethical considerations

King's College London minimal risk research ethical approval was obtained (MRA‐20/21–22,149).

## 3 RESULTS

### 3.1 Qualitative findings

**FIGURE 1:**
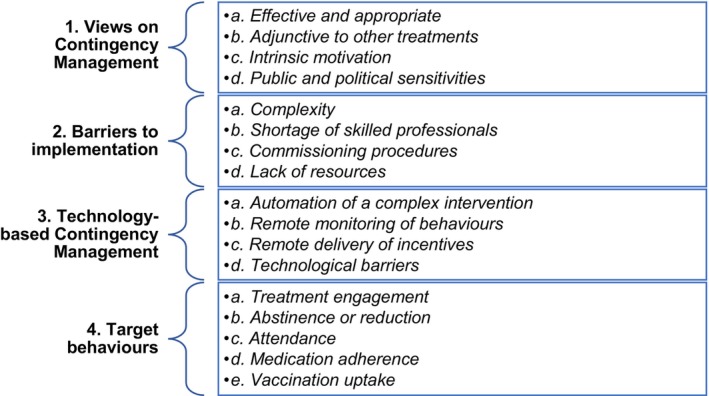
Overview of main and subthemes.

Four main themes and 17 subthemes were derived from the data (Figure 1). Although the expert group members differed in their professional roles, providing perspectives from both policy and clinical positions, they tended to express some similar views. Areas of disagreement and difference are highlighted where these occurred.

### 3.2 Views on contingency management

#### 3.2.1 Effective and appropriate

Most participants reported that CM is a theoretically grounded, scientifically evidence‐based, powerful and appropriate treatment approach for drug treatment and recovery. Participants demonstrated awareness of CM's evidence base, with some noting that for certain addictions, it is the only effective treatment available. Participants specifically highlighted CM's strong evidence of efficacy in improving appointment attendance and compliance with health interventions, arguing that it stands out among other psychosocial interventions.

‘The science behind it and the understanding of the sort of shaping and chaining or using it as part of a bigger picture often gets lost in that whole sort of bigger picture. But I undoubtedly think that I mean that's what to me addiction treatment, one of the most effective strategies is about finding positive rewards for alternative behaviours.’ (Clinician, P6)

However, participants acknowledged that, despite CM's scientific basis, it is often resisted when used for substance use behaviours, contrasted with its acceptance in other contexts, such as encouraging young people to undergo sexual health checks. They argued this disparity in acceptance highlights societal biases towards different populations. Despite this, participants highlighted that the importance lies in ensuring the targeted behaviour is meaningful and the intervention could lead to sustainable changes. One participant argued that while negative beliefs about CM might exist, a pragmatic approach is important — if it works, it should be used.

‘My approach kind of comes from a very harm reduction informed sort of ethos. In other words, it's pragmatism. It's what works. And I guess I've always been very taken by the fact that you know, certainly in some sense is that evidence around contingency management is so much stronger than other psychosocial interventions, and where you kind of weave amongst the dearth of evidence, contingency management does seem to kind of shine through.’ (Clinician, P12)

CM was also considered to be a cost‐effective approach, as the benefits of keeping people in treatment and improving their quality of life outweigh the minimal costs of providing incentives. Despite this, there is a lack of implementation in UK drug and alcohol services, with some participants expressing frustration over the barriers preventing its widespread adoption.

#### 3.2.2 Adjunctive to other treatments

Participants viewed CM as a powerful adjunctive tool that should be part of a comprehensive addiction treatment toolkit, as no single intervention works for everyone. Participants emphasised that CM should support and enhance existing treatment interventions, rather than replace them. It was seen as a valuable method to encourage adherence to other treatments and improve outcomes, especially when integrated with good keyworking^2^ and a strong therapeutic relationship.

‘We definitely have to sort of sell it as something to help people engage in treatment rather than actually being a treatment in itself.’ (Clinician, P8)

CM was viewed as a valuable approach for addressing behaviours that are often resisted, such as sexual health screenings, by providing incentives that can distract from negative stimuli. Participants emphasised that for people who are entrenched in substance use with complex needs, it is essential CM is part of a broader strategy that addresses the underlying reasons for substance use, such as trauma, with appropriate therapeutic support and evidence‐based psychological interventions.

#### 3.2.3 Intrinsic motivation

Participants generally felt that intrinsic motivation is crucial for behaviour change, with some arguing that CM is less effective without some level of intrinsic motivation from the individual. Perspectives differed on the strength of the relationship between intrinsic and extrinsic motivation. Some believed extrinsic motivation (i.e. incentives) can lead to intrinsic motivation, enhancing self‐efficacy and engagement over time, while others were sceptical, suggesting that extrinsic rewards do not always translate into intrinsic motivation. With that in mind, some participants believed that the degree of motivation required varies by behaviour, and some actions (such as health tests) do not require intrinsic motivation because the immediate benefits are clear and the actions are straightforward.

Participants emphasised that CM should target behaviours aligned with the individual's treatment goals and be part of a collaborative decision between the service user and clinician, not a standard, one‐size‐fits‐all approach. Participants were divided about using CM to encourage behaviours that clients are not ready to change. For some, they believed that CM would not be as beneficial if a client presented zero intrinsic motivation.

‘I still think there has to be a level of kind of intrinsic motivation that somebody has to get them to want to engage with it. And some people aren't motivated or they want to keep using and they're just not ready then.’ (Commissioner, P5)

Some participants argued that using CM when there is a lack of motivation is a moral issue: with CM becoming a ‘carrot and stick’ approach rather than a therapeutic tool. On the other hand, some argued that CM is less important for clients already motivated to change their behaviour and that it is paramount that clinicians encourage behaviour change even when clients are not fully invested, especially in high‐risk situations involving poly‐substance use.

‘I don't think it's unreasonable for me as a prescriber to be keen to support somebody, even somebody that's pre‐contemplative about their alcohol consumption or alcohol hazardous alcohol use and they don't want to change it.’ (Clinician, P15)

#### 3.2.4 Public and political sensitivities

Several participants, particularly policy professionals, considered societal and media perceptions of CM responsible for the lack of adoption, with CM being portrayed as a bribe rather than a legitimate therapeutic tool. One policy professional described how political sensitivities and apprehension around negative media attention resulted in national reports on CM implementation being ‘buried’.

‘It's a contentious area. And so basically, it's been a perfect storm against it … I don't think [name of government agency] ever produced the final report of the pilots they did, partly because of the political sensitivity at that time. I think [name of government agency] buried it … This whole area is politically contentious anyway.’ (Policy professional, P21)

### 3.3 Barriers to implementation

#### 3.3.1 Complexity

Participants reflected on the challenges of implementing CM interventions, with several highlighting some uncertainty about the mechanisms of action and the essential elements required for effective delivery. Several participants described UK addiction services as ‘unstructured’ and argued that the translation of evidence‐based interventions such as CM into clinical practice is difficult where keyworking often lacks the necessary clinical and psychological leadership or input.

‘Our treatment settings are relatively unstructured. There's an awful lot of key working which is unstructured key working as opposed to services being set up and clinically and psychologically led, such that people are delivering a defined intervention when they're working with a patient or a service user.’ (Clinician, P12)

#### 3.3.2 Shortage of skilled professionals

The current drug and alcohol workforce was described as overstretched and underskilled, which was argued to have a detrimental effect on the adoption of psychosocial interventions. Clinicians expressed concern about the lack of expertise within the workforce, with an over‐reliance on unqualified people and therefore limited implementation of quality psychosocial interventions. Some felt that the implementation of CM within UK drug and alcohol services faces barriers due to a lack of awareness and misunderstandings about CM, with some staff failing to grasp its theoretical basis and structured approach.

‘There's been a complete brain drain from the sector for Psychologists, who probably would be the profession that would be behind contingency management and at least would be the ones advocating that if you're gonna do it, you need to do with decent governance structures around now.’ (Clinician, P13)

#### 3.3.3 Commissioning procedures

Participants argued that CM is not being commissioned or recommended by commissioners, and therefore budgets to finance such interventions are not available. Changes in the commissioning process and tight funding complicate the introduction of CM, making it a challenging proposition amid other priorities. Commissioners argued that they would need to relocate funds, which might result in staff reductions or cuts to other services, and this creates a reluctance to adopt CM. One clinician described how the competitive tendering process in service commissioning discourages innovation and makes it difficult to implement new interventions like CM. This is exacerbated when commissioners lack an understanding of its benefits.

‘The services are continually changing, they are being retendered all the time, so there's little appetite I think sometimes for trying to do something that perhaps the service feels takes quite a lot of effort and that they may not even be running the service in a year or so.’ (Clinician, P17)

Commissioners argued that effective implementation requires top–down leadership, starting at the national level and filtering through regional teams to local authorities and service providers. One commissioner argued that there is a lack of adherence to clinical guidelines in general, and the current focus on harm reduction rather than abstinence and recovery ultimately affects the adoption of CM.

#### 3.3.4 Lack of resources

Participants blame general disinvestment in local authorities and public health for hindering the adoption of evidence‐based interventions, including CM. Participants argued that such disinvestment has resulted in a diminishing and overstretched workforce, with underfunding across local authorities, public health and drug and alcohol services making it difficult to stay updated with evidence‐based practice. Participants described how CM implementation requires substantial resources and administrative support, including managing incentives and ensuring proper documentation. There was a consensus that these logistical, administrative and workforce challenges hinder the widespread adoption of CM in clinical practice.

‘Our worlds have shrunk in terms of our teams and our workloads have grown. I think there are probably some sort of structural factors that are stopping this happening, and I think dis‐investment in local authorities, disinvestment in public health, grant and disinvestment in public health, capacity and disinvestment in drug and alcohol services are all playing a part in why it hasn't come about.’ (Commissioner, P1)

### 3.4 Technology‐based CM

#### 3.4.1 Automation of a complex intervention

Participants discussed leveraging technology to enhance the delivery of CM, emphasising its potential to streamline complex schedules and improve efficiency. They highlighted the feasibility of using technology‐based solutions to manage CM remotely, suggesting that this approach could facilitate a wider range of rewards and mechanisms. This technological shift was seen as imperative in reducing staff time and resources involved in CM delivery, thereby making it more scalable and accessible within current constraints.

‘We very successfully deliver all sorts of things remotely. And I think it opens up a whole new range of possible rewards and possible mechanisms because it's just the processes are easier if they are technology‐based.’ (Clinician, P4)

The development of an app to support staff in administering and managing CM interventions was proposed by several clinicians. With recognition of the challenges in training and deploying a workforce capable of delivering CM manually, clinicians suggested such an app would be able to automate certain aspects and simplify the process. Moreover, integrating CM into existing platforms, particularly those used for client management and goal tracking, was considered advantageous. Clinicians envisioned a system where CM activities could be seamlessly recorded and reinforced through automated notifications and data entries, enhancing the overall effectiveness of treatment interventions.

#### 3.4.2 Remote monitoring of behaviours

Participants expressed mixed views on the appropriateness and benefits of using technology, particularly remote drug testing, in treatment contexts. Some viewed it as entirely appropriate and beneficial for measuring behaviour change more effectively compared to traditional methods. They argued that technologies like oral fluid testing offer a less intrusive and more dignified alternative to urine testing, which is often seen as infantilising and punitive.

‘Moving stuff remotely has made us even just the removal of bloody urine testing, which is just you know, such a Freudian extension of potty training and having to rely on saliva testing has changed stuff for us. That is so different to you coming through the door and me as a grown man telling you, as a grown woman to go and piss in a little pot that I've given you.’ (Clinician, P4)

However, concerns were raised regarding the reliability and cost of the equipment needed for remote testing, particularly for behaviours like alcohol consumption that are challenging to measure remotely. There were also worries about the potential for clients to falsify results to gain rewards, indicating the need for robust verification methods. Many participants emphasised the importance of clear communication with clients about the purpose and procedures of remote testing to ensure informed consent and mitigate distrust. They recognised the potential benefits of clients monitoring their own behaviours, although they cautioned about the challenges of ensuring authenticity and reliability in remote monitoring.

#### 3.4.3 Remote delivery of incentives

Participants highlighted various perspectives on utilising technology to automate the delivery of incentives and enhance the effectiveness of CM interventions. While some recognised reward immediacy as essential and were enthusiastic about automating systems to deliver rewards immediately upon compliance with treatment behaviours, others expressed concerns about the resource intensiveness of such systems, both in terms of financial costs and logistical management. Ethical concerns were also flagged regarding the deceptive use of technology to deliver automated messages that simulate human interaction, with a few advocating for transparency to avoid misleading clients.

‘I feel quite strongly on this that it either has to be generated by a human being or there should be no pretence that it is generated by a human being. Because that is misleading clients.’ (Clinician, P10)

#### 3.4.4 Technological barriers

Participants voiced concerns about technological barriers that could hinder the effectiveness of digital interventions, particularly in CM for marginalised groups. Issues included low digital literacy, difficulties with technology and inconsistent access due to phone loss or changing numbers. There was scepticism about the uptake and efficacy of digital interventions compared to traditional face‐to‐face approaches, with concerns over low engagement rates seen in other online programmes. Several highlighted challenges of digital exclusion in substance use treatment, including the lack of suitable technology and internet access, especially among clients in less affluent areas. Additionally, device competency issues were noted, particularly for clients with cognitive impairments, with more stable clients typically better equipped to engage with digital interventions.

‘Most people have got smartphones but not everybody … it's a paradox that the most stable clients are the ones who are probably most logically capable to access things because they've got the equipment and the very often got jobs. And they are geared up for it.’ (Clinician, P9)

### 3.5 Target behaviours

#### 3.5.1 Treatment engagement

Participants identified poor treatment engagement and retention as significant challenges within the substance use treatment sector. They highlighted difficulties in retaining clients and high dropout rates, emphasising that engagement and retention are crucial for effective outcomes. There was a general acceptance among participants towards using CM to encourage engagement. Using CM to encourage treatment initiation was proposed by one participant, in recognition of the need to reach those not accessing treatment services.

‘The people that are dying from substance misuse are people outside of treatment, and they're the ones that we desperately need to get into treatment. They are the hardest on engagement and compliance.’ (Clinician, P4)

However, concerns were raised that CM might only achieve surface‐level engagement without fostering meaningful therapeutic interaction. Clinicians stressed the importance of offering substantial therapeutic support to ensure that attendance translates into genuine client progress and recovery. Similarly, contrasting views were presented regarding the sequence of using CM: some suggested that the initial focus should be on getting individuals into treatment, viewing this as a critical first step before deeper therapeutic work can commence. Others cautioned against incentivising engagement in programs lacking meaningful content, advocating instead for incentives that promote engagement in constructive, recovery‐oriented activities.

#### 3.5.2 Abstinence or reduction

CM targeted at a reduction or cessation of substance use elicited a spectrum of opinions regarding its goal orientation and the sustainability of its interventions. While many participants supported using CM to promote reductions or abstinence from substances, concerns were voiced that this might inadvertently reinforce a value judgement in favour of abstinence over harm reduction or moderated use, underscoring the importance of tailoring interventions to align with individual client goals. Participants emphasised the importance of addressing the functional aspects of substance use and promoting alternative behaviours alongside CM interventions. This approach was seen as crucial for enhancing the effectiveness and sustainability of CM in achieving long‐term behavioural change.

‘We should use it in ways that are meaningful to them. If it's what a client wants to talk about. But the other thing, drug use for so many of our clients is functional. Before we take away any behaviour we would need to be paying some attention to functional equivalence. I've yet to see it create lasting or enduring change.’ (Clinician, P10)

#### 3.5.3 Attendance

Participants emphasised that non‐attendance is a significant issue in substance use treatment settings, and while the majority expressed support for using CM to improve attendance, concerns were raised about ensuring that the attendance translates into meaningful therapeutic engagement. Several participants cautioned against using CM purely for attendance without considering the quality and impact of the interventions provided during these visits.

‘Oh, it's 100% appropriate. That's where I would use it. You know it's great if you attend here, but what are you doing when you're actually here is the key. But if you don't do anything worthwhile when they're in the building, it's all a waste of time just getting in the door.’ (Clinician, P6)

Several participants also expressed support for using CM to encourage attendance at mutual aid groups, emphasising the beneficial impact a supportive environment with peer interaction can have on recovery.

#### 3.5.4 Medication adherence

Participants reported medication adherence as a widespread issue across healthcare: consuming significant staff resources and impacting service efficiency. CM was considered a valuable tool to address this issue, either supporting optimal dosing and treatment outcomes or adherence to medications with unpleasant side effects.

‘Theoretically in terms of acceptability, I think it would help. I think particularly where some medications can be horrible and the patient's experience of that isn't particularly nice.’ (Commissioner, P5)

However, not all viewed CM for medication adherence as appropriate for routine standard practice. Several suggested it should be selectively applied to specific target populations rather than universally adopted. Concerns were raised regarding political sensitivities and media scrutiny surrounding the use of financial incentives for medication adherence, indicating a need for careful consideration of public perception.

#### 3.5.5 Vaccination uptake

The majority of participants considered it acceptable to use CM to incentivise vaccination uptake among clients. There was a consensus that CM in this context does not necessarily aim to change long‐term behaviour, but rather to facilitate a specific health‐promoting action that might otherwise be neglected due to chaotic life circumstances. The majority viewed CM as appropriate and effective for encouraging vaccinations against hepatitis B or hepatitis C, recognising the potential public health benefits of reducing transmission and improving individual health outcomes.

‘So like blood borne virus vaccinations and ethically I can really square that with my head because of the public health benefit.’ (Clinician, P9)

## 4 DISCUSSION

Research on CM has grown exponentially over the last decade, with remote technologies being leveraged to expand the reach and scope of these interventions [25]. This coincides with technological innovations in the delivery of healthcare interventions in general, due to the COVID‐19 pandemic and public health control measures [27]. This qualitative exploration of addiction specialists' perspectives on CM and the remote delivery of these interventions seems timely and also essential to optimise and facilitate its wider introduction and competent delivery.

While specialists widely acknowledged CM as an effective, scientifically grounded and powerful tool in a comprehensive addiction treatment toolkit, they identified significant barriers impeding its implementation, including the complexity of these interventions, lack of awareness and misunderstandings about CM's evidence base and theoretical underpinnings, and under‐resourced addiction services. Consistent with previous work, societal and media perceptions of CM have a destructive impact on attitudes towards these interventions, with political sensitivities and apprehension around negative media attention [29]. Attitudes towards the use of basic behavioural principles to reinforce behaviour change among those with neurodevelopmental conditions, such as autism and those with substance use disorder are hugely contrasted [30]. Even within the field of substance use, our data suggest that CM used to address behaviours of public health relevance might be considered more palatable and ethical.

Concerns have been raised about the ethical and clinical risks of pushing clients towards behaviours they are not ready for, with CM potentially becoming a ‘carrot and stick’ approach rather than a therapeutic tool. These concerns have previously been highlighted in the literature, albeit in studies from over a decade ago and predominantly from the US, where the treatment system differs from that in the UK [21, 22, 23]. However, awareness and experience with CM are linked to more positive perceptions of these interventions, with more experienced clinicians tending to support its use [21, 22, 31, 32]. This may also explain the contrast between our findings and those of studies that report greater concerns about CM's application among clinicians with limited awareness or experience [29]. While some scepticism remains, a pragmatic stance prevails in the literature – if CM is effective, it should be used [22].

While CM is considered to be a valuable tool in encouraging adherence to treatments and improving outcomes, specialists emphasise the importance of integrating these interventions with good keyworking and a strong therapeutic relationship. This is imperative in improving motivation, participation in treatment and increasing the likelihood of recovery [33]. CM delivered in isolation and not as part of a comprehensive therapeutic toolkit is seen as insufficient for people with high levels of substance use given the complex nature of addiction. CM must be part of a broader strategy that addresses the underlying reasons for substance use, such as trauma, with appropriate therapeutic support and evidence‐based psychological interventions.

Several specialists alluded to the philosophy of substance use treatment, focusing on the role of intrinsic motivation and how CM might influence it. Some argued that extrinsic motivation, such as rewards in CM, can eventually lead to intrinsic motivation, enhancing self‐efficacy and long‐term engagement. However, others were sceptical, suggesting that extrinsic rewards may not always lead to intrinsic motivation and could potentially undermine it. While this debate persists in the literature, evidence indicates that CM does not diminish intrinsic motivation [34] and CM can in fact promote natural reinforcers by encouraging behaviours that lead to lasting positive outcomes, such as better health and stronger social connections [35].

There were concerns that CM's focus on abstinence may conflict with harm‐reduction approaches central to many current services. This tension reflects previous findings that CM does not always align with the broader philosophy of harm reduction used in many substance use treatment settings [22]. Traditional CM protocols, with their escalating incentives and resets for abstinence, aim to prevent relapse rather than prioritise harm reduction. Harm reduction strategies remain essential for mitigating the broader negative impacts of SUD [36]. While the evidence consistently supports CM's effectiveness in promoting abstinence [2, 3, 37], CM protocols designed to improve attendance, medication adherence and vaccination uptake among those in treatment for SUD are also consistent with harm reduction agendas and have yielded medium–large effects [9, 10, 11, 38]. We must also recognise that in practice, while not all clients will achieve abstinence, a decrease in the frequency of substance use is clinically meaningful and should be recognised. We ought to consider how CM protocols can be adapted to accommodate reductions in substance use, such as utilising tests that can detect reductions in use or minimising the impact of resetting incentives in response to a positive result [39].

Digital CM was considered to be advantageous, offering remote monitoring and easier delivery of incentives: increasing access and reducing treatment barriers [12]. However, important concerns were raised about digital literacy, technological challenges and financial limitations. Specialists emphasised the importance of balancing the benefits of digital interventions with the need to ensure authenticity, reliability and inclusivity. Competency in using devices was also a concern, particularly for clients with cognitive impairments or limited experience with technology. Specialists noted that more stable clients, who typically have better access to and proficiency with the necessary equipment, are often best positioned to engage with digital interventions. Nonetheless, there was broad recognition of the need to integrate technology into treatment practices in today's digital era. With smartphone ownership reaching 85% among the SUD treatment population [40], leveraging technology could offer a viable and widely accepted approach [41].

This qualitative study has yielded invaluable insights into perceptions of CM held by policy and clinical experts within the addiction field. It provides an in‐depth understanding of why such a strong evidence base for its efficacy is not being widely implemented in clinical practice. However, this study is not without its limitations. While online interviews were essential due to COVID‐19 restrictions and allowed for greater flexibility and accessibility, they may have influenced the depth and quality of the data collected. The virtual format could have limited the ability to build rapport with participants, potentially leading to less detailed or open responses. Furthermore, providing participants with information on the intervention in the form of a summary sheet prior to the interviews could have introduced bias in their responses. While the team deemed it important to ensure participants had an opportunity to reflect on their position prior to the interview, reducing likely discourse that might be influenced by the researcher's own subjectivity, this pre‐interview exposure to information might have affected the objectivity of the data collected and may have limited the study's ability to capture more spontaneous or unbiased perspectives. While efforts were made to reduce the impact of researcher bias, preconceptions and experience of conducting and evaluating CM interventions are likely to have impacted the interpretation of the data. Finally, it is important to note that addiction specialists working within local services with different treatment and funding models may have different perspectives, and therefore future research evaluating implementation in local contexts is important.

## 5 CONCLUSION

The UK government's 10‐year drug strategy emphasises the importance of implementing evidence‐based approaches to reduce substance use harms [42]. The strategy advocates for the creation and evaluation of technological interventions that offer innovative and evidence‐based approaches to address problems associated with substance use. While there are barriers and ethical issues to implementing CM, its effectiveness, cost‐efficiency and potential to improve treatment outcomes make it a valuable component of addiction treatment strategies. Addiction specialists' advocacy efforts and collaboration with academic–industry partnerships are essential to advance the digital CM space and improve outcomes for individuals with SUD [43]. While existing evidence supports using digital CM to encourage treatment‐related behaviours [9, 14, 16, 44], concerns remain about the practicality of delivering CM via technology. This underscores the need for further research to explore its feasibility as a cost‐effective solution to enhance access to this evidence‐based intervention in UK treatment services that face significant resource constraints.

## AUTHOR CONTRIBUTIONS

Each author certifies that their contribution to this work meets the standards of the International Committee of Medical Journal Editors.

## FUNDING INFORMATION

The research reported in this publication was financially supported by the Society for the Study of Addiction as part of CAG's post‐doctoral development award. The funders had no role in the study design and data analysis. The findings and conclusions in this publication are those of the authors and do not necessarily represent the views of the funder.

## CONFLICT OF INTEREST STATEMENT

JS is a researcher and clinician who has chaired/contributed to guidelines on policy and practice and has led studies of the impact of changes in practice. He has also worked with pharma and technology companies to investigate new or improved medications, devices or programs to explore potential improvements to treatment, including (past 3 years) with the app‐developer CMI. However, none are related directly to the areas reported in this study. NM has received, through her university, King's College London, research funding from Mundipharma Research Ltd. JS, NM and CAG are involved in research projects which, through sub‐contracts, use the capability of the app‐development company CMI.

## Data Availability

Research data are not shared.

## Endnotes

^1^
Participant details have been omitted to preserve anonymity.

^2^
A keyworker is a practitioner responsible for delivering and reviewing a client's care plan, typically through regular meetings. They are part of a multidisciplinary team and ensure the care plan is adjusted as needed. Keyworking involves building a therapeutic relationship and creating both immediate and long‐term care plans.

## APPENDIX A TOPIC GUIDE

**Table:**

Introduction	Introduction: Obtain participant characteristic data
Contingency management	General views on CM interventions: Positive/negative
	Awareness, familiarisation, experience
	Effectiveness/usefulness of CM as a behaviour change intervention
	Barriers to implementing CM
	Appropriateness of tangible/social incentives/target behaviours
	Impact on clients
	Impact on the relationship between client and treatment provider
CMs place UK drug and alcohol treatment services	Issues of non‐compliance/engagement with treatment
	Factors affecting client's compliance with their treatment
	CM targeted at: Attendance at the clinic/medication adherence/reduction in substance use/uptake of needle exchange/ uptake with routine tests and vaccinations.
	Population groups that would benefit most from CM
	Treatment service's willingness to implement CM/requirements
Remote CM	Views on the use of technology to expand the reach of treatment/deliver CM
	Advantages of delivering CM remotely/feasibility of implementation
	Concerns about remote CM
	Monitoring target behaviours remotely
	Delivering financial incentives remotely (study debit cards)
Future mCM	Clinical priorities/needs. Need for CM?
	Target behaviours that need to be prioritised
	Client group(s) that are most in need or particularly vulnerable and might benefit the most from CM
	Expectations of future CM interventions
	Difficulties and barriers to delivering CM remotely
	Views on the appropriate treatment duration/intensity/reinforcer needed to promote and sustain desirable behaviour change

Abbreviations: CM, Contingency management; mCM, mobile telephone‐delivered contingency management.

## APPENDIX B CODING FRAMEWORK

**Table:**

Categories		Codes and frequencies
Views on contingency management		Adjunct to other treatment (6)
		Appropriate (5)
		Complexity (1)
		Context (2)
		Effective (20)
		Impact of CM (15)
		Incentives (17)
		Intrinsic motivation (9)
		Negative side effects (4)
		Selective (1)
		Sustainability (4)
		Theory driven (3)
		Therapeutic alliance (19)
		Tokenistic (1)
		Transactional (1)
		Unethical (4)
		Willingness to implement (12)
Barrier to implementation		Complex intervention (5)
		Costly (12)
		Lack of awareness (7)
		Local evidence (2)
		Moral objections (19)
		Not commissioned (12)
		Not in treatment (1)
		Practical issues (16)
		Unconventional approach (4)
		Unskilled workforce (10)
Strategies to overcome barriers		Awareness & training (9)
		Embed in treatment systems (9)
		Increase resources (2)
		UK pilot projects (3)
Target behaviours/populations		Abstinence (22)
		Attendance (13)
		Medication adherence (14)
		Mutual aid groups (5)
		Other health‐related behaviours (6)
		Relapse prevention (1)
		Routine health appointments (3)
		Simple behaviours (1)
		Treatment engagement (20)
		Vaccination adherence (12)
		Wider treatments (2)
Technology‐based contingency management		Allow personalisation (6)
		Automated (3)
		Challenges (11)
		Current in the digital age (11)
		Delivering incentives remotely (14)
		Digital exclusion (19)
		Enhances access (10)
		Evidence base (1)
		Lacks personal contact (9)
		Monitoring behaviour remotely (21)
		Privacy (10)
		Reliable (1)
		Responsibility (3)
		Scalable (4)
		Tackles resourcing issues (9)
		Unacceptable (1)
